# CD64-directed microtubule associated protein tau kills leukemic blasts *ex vivo*

**DOI:** 10.18632/oncotarget.11568

**Published:** 2016-08-24

**Authors:** Radoslav Mladenov, Dmitrij Hristodorov, Christian Cremer, Gerrit Gresch, Elena Grieger, Lea Schenke, Diana Klose, Manal Amoury, Mira Woitok, Edgar Jost, Tim H. Brümmendorf, Rolf Fendel, Rainer Fischer, Christoph Stein, Theo Thepen, Stefan Barth

**Affiliations:** ^1^ Fraunhofer Institute for Molecular Biology and Applied Ecology, Aachen, Germany; ^2^ Department of Experimental Medicine and Immunotherapy, Institute of Applied Medical Engineering, University Hospital, RWTH Aachen University, Aachen, Germany; ^3^ Department of Haematology and Oncology (Internal Medicine IV), RWTH Aachen University Hospital, Aachen, Germany; ^4^ Institute of Molecular Biotechnology (Biology VII), RWTH Aachen University, Aachen, Germany; ^5^ South African Research Chair in Cancer Biotechnology, Institute of Infectious Disease and Molecular Medicine (IDM), Department of Integrative Biomedical Sciences, Faculty of Health Sciences, University of Cape Town, South Africa

**Keywords:** immunotherapy, myeloid leukemia, cytolytic fusion proteins, Fc-gamma receptor (CD64), microtubule associated protein tau (MAP)

## Abstract

Fc gamma receptor I (FcγRI, CD64) is a well-known target antigen for passive immunotherapy against acute myeloid leukemia and chronic myelomonocytic leukemia. We recently reported the preclinical immunotherapeutic potential of microtubule associated protein tau (MAP) against a variety of cancer types including breast carcinoma and Hodgkin's lymphoma. Here we demonstrate that the CD64-directed human cytolytic fusion protein H22(scFv)-MAP kills *ex vivo* 15–50% of CD64^+^ leukemic blasts derived from seven myeloid leukemia patients. Furthermore, in contrast to the nonspecific cytostatic agent paclitaxel, H22(scFv)-MAP showed no cytotoxicity towards healthy CD64^+^ PBMC-derived cells and macrophages. The targeted delivery of this microtubule stabilizing agent therefore offers a promising new strategy for specific treatment of CD64^+^ leukemia.

## INTRODUCTION

Leukemia is the most common hematologic malignancy, with 54,270 new cases in the USA in 2015, representing a 14.4% increase over 2012. The most common forms of leukemia are acute myeloid leukemia (AML) representing 40% of all cases, followed by chronic lymphocytic leukemia (CLL) and chronic myeloid leukemia (CML) [1–3].

Treatment strategies for myeloid leukemia include chemotherapy, radiotherapy and immunotherapy, with current research focusing on antibody-based targeted immunotherapy because of its versatility and advantages in terms of production, regulatory compliance and pharmaceutical logistics [4]. Several molecules overexpressed on the surface of AML cells have been investigated as targets for antibody-based therapy, including CD33, CD47, C-type lectin, CD64, CD123 and most recently CD89 [5–10].

CD33 is the most common target for AML therapy. The antibody-drug conjugate (ADC) gemtuzumab ozogamicin targets this molecule, and showed promising efficacy as a monotherapy (overall response rate of ∼30%). However, is use as a supplement to conventional chemotherapy achieved no survival advantage but caused higher toxicity in phase III trials, leading to the withdrawal of approval in 2010 [11, 12]. Alternative targeting agents include immunotoxins (ITs) and targeted fully human cytolytic fusion proteins (hCFPs). In both cases, selectivity is achieved by an antibody derivative such as a single chain variable fragment (scFv). Conventional ITs use bacterial toxins to induce apoptosis, such as truncated *Pseudomonas aeroginosa* exotoxin A (ETA’) [13], whereas hCFPs are equipped with human pro-apoptotic effectors such as granzyme B, granzyme M or angiogenin [8, 14, 15]. The main disadvantages of ITs are the immunogenicity of the non-human effectors and off-target effects such as hepatotoxicity and vascular leakage, which have limited their performance in clinical trials [16]. In contrast, hCFPs are fully human constructs and the risk of immunogenicity is much lower [17].

We previously reported the identification of a novel cytolytic effector protein known as microtubule-associated protein tau (MAP) which promotes the assembly of the mitotic spindle [18–20]. MAP binds to microtubules via microtubule-binding repeats and enhances the stability of microtubule polymers [21–23]. Native MAP is regulated by phosphorylation at two sites to induce its dissociation and allow microtubule disassembly after mitosis, so the cytotoxic version of MAP was mutated to remove these sites (S156A and S204A) [18]. Consequently, the modified MAP binds irreversibly to microtubules and interrupts their normally dynamic behavior, ultimately leading to the induction of apoptosis [24].

The modified MAP protein kills proliferating EGFR^+^ cancer cells, EpCAM^+^ carcinoma cells, AChR^+^ rhabdomyosarcoma cells and CD30^+^ lymphoma cells when fused to appropriate targeting components [18–20, 25]. Most recently, the CD64-targeting construct H22(scFv)-MAP was shown to eliminate pro-inflammatory M1 macrophages but not the anti-inflammatory M2 population following intradermal administration to the chronically inflamed skin of transgenic mice expressing human CD64 [26]. The latter study also revealed that H22(scFv)-MAP kills rapidly-proliferating pro-leukemic/monocytic HL-60 cells in a dose-dependent manner, indicating a potential for anti-leukemic activity.

We therefore tested H22(scFv)-MAP against primary cells isolated from seven untreated leukemia patients. Our *ex vivo* data confirmed the targeted pro-apoptotic activity of H22(scFv)-MAP and suggest it should be developed further as an immunotherapeutic candidate for the treatment of leukemia.

## RESULTS

### Phenotyping primary cells from leukemia patients

**Figure 1:**
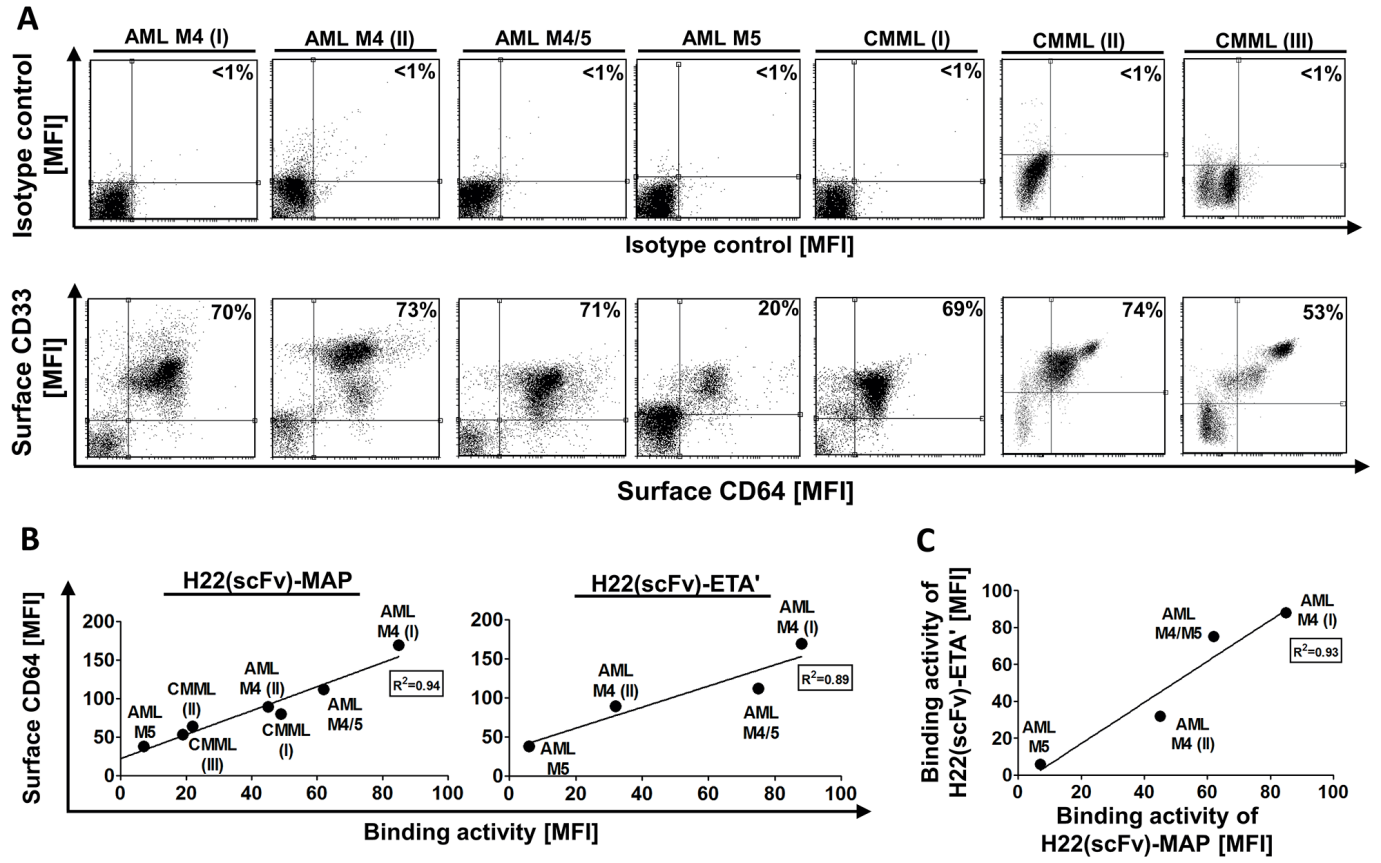
Characterization of the CD64^+^ leukemic blasts and H22(scFv)-MAP binding Blood samples were obtained from seven untreated patients diagnosed with different forms of leukemia (Table 1). **A.** The proportion of primary CD33^+^ CD64^+^ leukemic blasts from seven patients is compared directly to the isotype control. Percentiles indicate the proportion of double positive cells from all measured events. **B.** The ability of purified H22(scFv)-MAP to bind the leukemic cells *in vitro* was tested by flow cytometry. The binding activity of H22(scFv)-MAP (left) and H22(scFv)-ETA’ (right) correlate to the estimated CD64^+^ level. **C.** Comparative binding activities of both CD64-targeting fusion proteins. Abbreviations: MFI, mean fluorescence intensity.

Double staining for CD33 and CD64 indicated that blood samples from both AML M4 patients, the AML M4/M5 patient and two of the three CMML patients yielded ∼70% CD33^+^ CD64^+^ blasts (Table 1). The other CMML sample contained 53% CD33^+^ CD64^+^ blasts, whereas the sample from the AML M5 patient yielded only 20% CD33^+^ CD64^+^ blasts (Figure 1A).

**Table 1:** Characteristics of the leukemia patients

Parameter	Number						
**Patient designation**	AML M4 (I)	AML M4 (II)	AML M4/M5	AML M5	CMML (I)	CMML (II)	CMML (III)
**Age**	51	78	81	74	91	79	n.i.
**Gender**	Female	Male	Male	Male	Female	Male	n.i.
**FAB subtype**	AML M4 (AMML)	AML M4 (AMML)	AML M4/M5	AML M5	CMML-2	CMML-2	CMML-2
**WBC (G/L)**	80.7	88.7	113.4	104.9	105.7	4.9	n.i.
**% Monocytes**	3	1	44	6	9	23.6	n.i.
**Hemoglobin (g/L)**	91	116	10	83	98	119	n.i.
**Platelet count (G/L)**	28	45	134	128	64	258	n.i.
**Previous treatment**	No	No	No	No	No	No	No
**CD64^+^ CD33^+^ (%)**	70	73	71	20	69	74	53
**CD64^+^ cells (%)**	70	73	73	23	70	74	53

FAB = French-American-British classification; n. i. = no information.

### Binding of H22(scFv)-MAP to CD64^+^ target cells

H22(scFv)-MAP bound to CD64^+^ blasts from both AML M4 patients, the AML M4/M5 patient and all three CMML patients (Supplementary Figure S1A). H22(scFv)-ETA’ showed an almost identical binding profile, but was only tested against the samples from AML patients (Supplementary Figure S1B). Both constructs showed very low binding activity to the leukemic blasts from the AML M5 patient even though as stated above the sample contained ∼20% CD64^+^ CD33^+^ cells. As expected, the detection antibody alone and a CD30-specific non-binding control (Mock-MAP) did not bind to the isolated leukemic blasts. The binding activity of H22(scFv)-MAP and H22(scFv)-ETA correlated to the surface expression levels of CD64 on the blasts (Figure 1B) and there was also a strong correlation in binding properties when these constructs were compared (Figure 1C).

### H22(scFv)-MAP induces apoptosis specifically in leukemic blasts

**Figure 2:**
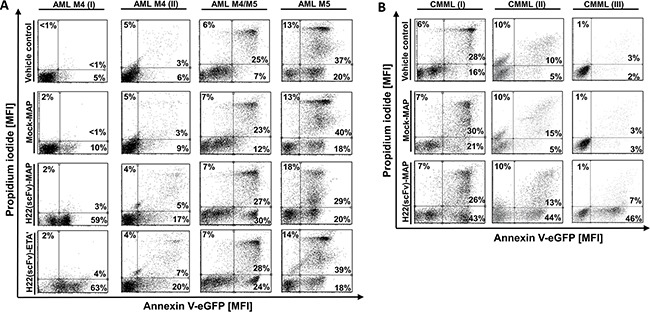
H22(scFv)-MAP induces apoptosis in CD64^+^ leukemic blasts *ex vivo* Leukemic blasts isolated from four AML patients **A.** and three CMML patients **B.** were treated *ex vivo* with 200 nM H22(scFv)-ETA’, H22(scFv)-MAP or Mock-MAP for 12 h. The leukemic cells were stained with Annexin V–eGFP and PI. Dot blots (ungated) show the specific pro-apoptotic effect of the H22(scFv)-MAP compared to Mock-MAP and H22(scFv)-ETA’. The experiment was carried out in duplicate.

**Figure 3:**
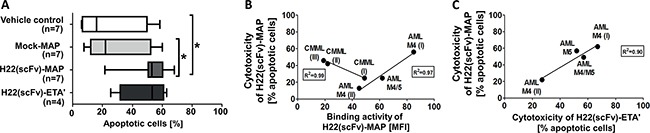
H22(scFv)-MAP is cytotoxic towards CD64^+^ leukemic blasts *ex vivo* **A.** Quantitative analysis of the dot blots in Figure 2 representing the proportion of cells undergoing apoptosis (the sum of early (Annexin V^+^/PI^−^) and late (Annexin V^+^/PI^+^) apoptotic cells) in the different experimental groups. Values are presented as box and whiskers plots with the interquartile range and minimum/maximum values indicated by the error bars. Each measurement was taken in duplicate. Statistical analysis was carried out using the non-parametric one-way ANOVA test (Kruskal-Wallis test by ranks): *p ≤ 0.05. **B.** Cytotoxicity of H22(scFv)-MAP plotted against its binding activity on leukemic blasts. **C.** Correlation between the cytotoxicity of the CD64-targeting fusion proteins. Abbreviations: MFI, mean fluorescence intensity.

Having confirmed the specific binding of H22(scFv)-MAP to CD64^+^ leukemic blasts, we tested the CD64-specific cytotoxicity of H22(scFv)-MAP, Mock-MAP and H22(scFv)-ETA’ by staining with Annexin V-eGFP (AV) and propidium iodide (PI) to detect cells undergoing apoptosis. We have shown in previous studies that 200 nM is a sufficient dose of hCFP or IT to achieve the receptor-specific induction of apoptosis within 12 h [7, 8, 14, 15]. We therefore used the same dose for the constructs tested in this study. First we ascertained the background frequency of apoptosis in each sample using a vehicle control, i.e. the buffer formulation but no active fusion protein. In most samples, 5–15% of cells underwent apoptosis spontaneously, but the frequency was higher for the AML M4/M5 (∼32%), AML M5 (57%) and CMML (I) (44%) samples (Figure 2). Importantly, the background frequency of apoptosis did not increase in the presence of Mock-MAP, confirming that MAP is not cytotoxic in the absence of specific binding. In contrast, the proportion of early-apoptotic (AV^+^/PI^−^) cells increased substantially in six of the samples when the leukemic cells were treated with H22(scFv)-MAP (Figure 3A). The AML M5 blasts exhibited the highest level of background cell death, which in combination with low CD64 profile resulted in no response to H22(scFv)-ETA’ or H22(scFv)-MAP treatment (Figure 2A). The pro-apoptotic effect of H22(scFv)-ETA’ correlated to that of H22(scFv)-MAP in the samples from the four AML patients (Figure 3C). Interestingly, there was a correlation between the binding activity and cytotoxicity of H22(scFv)-MAP in the AML samples, but in the CMML samples there was an inverse relationship between binding activity and cytotoxicity (Figure 3B).

### H22(scFv)-MAP was safer than paclitaxel against primary PBMCs and TAMs

**Figure 4:**
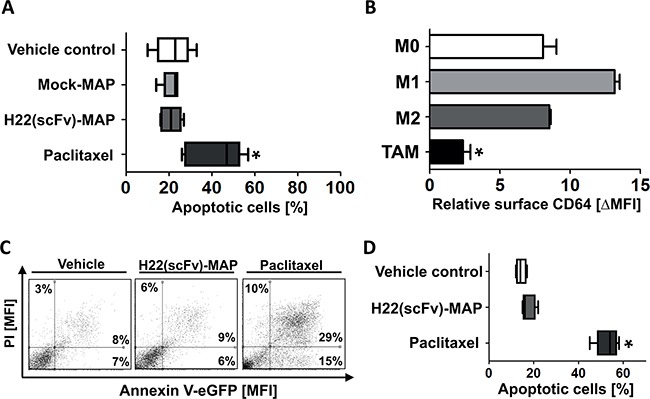
H22(scFv)-MAP shown no cytotoxicity towards healthy PMBC-derived cells **A.** Quantitative analysis of the proportion of healthy PMBC-derived cells undergoing apoptosis (the sum of early (Annexin V^+^/PI^−^) and late (Annexin V^+^/PI^+^) apoptotic cells) in the different experimental groups. Dot blots are shown in Supplementary Figure S2. **B.** TAMs express significantly lower amounts of CD64. The levels of CD64 on differently polarized, healthy PBMC-derived macrophages (n=3 for M0/1/2 and n=5 for TAM) were assessed by flow cytometry. **C.** Representative dot blots (ungated) confirm that TAMs are resistant to the CD64-directed MAP hCFP but not to the nonspecific cytostatic agent paclitaxel. **D.** Quantification of TAM apoptosis (n=5 PBMC donors) in the different experimental groups. Values are presented as box and whiskers plots with the interquartile range and minimum/maximum values indicated by the error bars. Each measurement was taken in duplicate. Statistical analysis was carried out using the non-parametric one-way ANOVA test (Kruskal-Wallis test by ranks): *p ≤ 0.05.

Peripheral blood mononuclear cells (PBMCs) from healthy blood donors were used to investigate nonspecific toxicity of H22(scFv)-MAP. Figure 4A confirms that neither Mock-MAP nor H22(scFv)-MAP are cytotoxic towards freshly-isolated PBMCs, whereas paclitaxel is potently cytotoxic at same concentration of 200 nM (dot blots in Supplementary Figure S2). The isolated PBMCs were then polarized to different macrophage phenotypes: M0, M1, M2, and tumor-associated macrophages (TAMs) (Supplementary Figure S4). The TAMs expressed much lower levels of CD64 than the other phenotypes (Figure 4B), resulting in weak H22(scFv)-MAP binding activity (Supplementary Figure S1C). Accordingly, CD64^low^ TAMs showed strong resistance towards H22(scFv)-MAP treatment, whereas paclitaxel induced apoptosis in all the healthy cells we tested (Figures 4C and 4D).

## DISCUSSION

Our experiments showed that H22(scFv)-MAP is suitable for the targeted elimination of CD64^+^ leukemic blasts from patients with different leukemic subtypes. The advantages of CD64 as a target for the delivery of cytotoxic effector molecules include its rapid internalization, which reduces the likelihood of off-target effects during hCFP therapy [8, 27, 28]. The therapeutic potential of H22(scFv)-MAP was recently demonstrated against inflammatory CD64^high^ macrophages (M1 subtype) in the context of chronic cutaneous inflammation [26]. The M1 population is thought to be beneficial in a tumor environment (including solid tumors and circulating tumor cells) but this environment is overwhelmed by anti-inflammatory signals, and type-II cytokines (e.g. IL-4, IL-10 and IL-13) tend to dominate (Supplementary Figure S3) [29–32]. The anti-inflammatory cytokines inhibit the M1 phenotype (CD64^high^) and induce alternative polarized macrophages (CD64^low^ M2/TAM subtype), which according to our observations are resistant to H22(scFv)-MAP (Figure 4) [26, 30, 33]. It is therefore anticipated that the homeostasis of the healthy CD64^+^ myeloid-derived cell population is spared by hCFP treatment because cells strictly representing the M1 subtype are sensitive [26, 33]. Furthermore, the co-expression of CD64 and CD33 by 50–70% of the leukemic blasts in most of our samples confirms their malignant nature. The binding of H22(scFv)-MAP in most samples demonstrated the efficient recognition of leukemic blasts. The exception was AML M5, which had only ∼20% CD64^+^ CD33^+^ blasts, and this sample also failed to respond to the *ex vivo* treatment with H22(scFv)-MAP. Interestingly, the cytotoxic potency of the hCFP correlated directly with the abundance of CD64^+^ in AML blasts, but there was an inverse correlation in the CMML blasts. This may reflect the distinct maturation stages of the acute and chronic myeloid blasts [34], which could result in the CD64-delivered cargo undergoing different forms of intracellular processing. Notably, our data suggest that H22(scFv)-MAP has the ability to kill both dim and bright CD64^+^ cells.

Cytostatic agents (e.g. paclitaxel) that disrupt microtubule dynamics are already used for chemotherapy against diverse forms of cancer [35–37]. These act in a similar way to MAP by stabilizing polymerized microtubules, blocking mitosis and ultimately leading to cell cycle arrest and apoptosis [38]. However, in line with our observations, the nonspecific delivery of taxanes causes off-target effects, including life-threatening toxicity towards healthy cells [39]. This was overcome by developing microtubule-blocking ADCs such as the FDA-approved trastuzumab emtansine (anti-HER2) and brentuximab vedotin (anti-CD30) [40–42]. Using the rationale for targeted microtubule-blocking therapy, H22(scFv)-MAP demonstrated the ability to bind CD64^+^ primary cancer cells specifically and kill them efficiently. Furthermore, no untargeted cytotoxicity was observed by treating the same cells with a non-binding Mock-MAP control. Our *ex vivo* data revealed that six of seven leukemia patients responded to the CD64-directed hCFP and its cytotoxic efficacy was similar to that of hCFPs based on granzymes or angiogenin [8, 14, 15].

In summary, H22(scFv)-MAP is an efficacious immunotherapeutic candidate that could be developed as a post-remission treatment for CD64^+^ hematological diseases, or combined with standard chemotherapy to improve prognosis. The development of novel immunotherapeutic strategies based on hCFPs is necessary because the FDA has withdrawn its approval for gemtuzumab ozogamicin, the only ADC approved for the treatment of AML, due to its lack of efficacy and the off-target toxicity revealed during phase III clinical trials [43].

## MATERIALS AND METHODS

### Patent samples and cell cultivation

Peripheral blood was collected from either untreated leukemia patients (diagnosed according to FAB classifications and clinical parameters as shown in Table 1) or healthy individuals after written informed consent was received and with the approval of the Clinical Research Ethics Board of the University of Aachen. PBMCs were isolated by density gradient centrifugation using Biocoll separating solution (BioChrom GmbH, Berlin, Germany). After three washes in phosphate buffered saline (PBS), the cells were cultured in standard RPMI 1640 medium (Invitrogen, Thermo Fisher Scientific, Waltham, MA, USA) supplemented with 10% (v/v) inactivated human serum (BioChrom), 50 μg/ml penicillin and 100 μg/ml streptomycin (Gibco, Thermo Fisher Scientific) at 37°C, 5% CO_2_ and 100% humidity.

### Production of the recombinant H22(scFv)-MAP hCFP

Recombinant H22(scFv)-MAP was expressed in *Escherichia coli* BL21 (DE3) cells using the protocol for periplasmic stress expression in the presence of compatible solutes as previously described [26]. Briefly, bacteria were grown after transformation to an optical density (OD) of 1.6 followed by stress induction with 500 mM D-sorbitol, 10 mM betaine monohydrate and 4% (w/w) NaCl. After incubating for 30 min at 26°C with agitation (180 rpm), protein expression was induced by adding 2 mM isopropyl β-D-1-thiogalactopyranoside (IPTG). Bacteria were harvested 18 h after induction by centrifugation (4000 × *g*, 10 min, 4°C) and frozen at −80°C overnight. The frozen pellet was resuspended in preparation buffer (75 mM Tris-HCl, 300 mM NaCl, 5 mM DTT, 10 mM EDTA, 10% (v/w) glycerol, pH 8.0) containing a complete protease inhibitor cocktail (Roche, Penzberg, Germany) at 4°C and sonicated five times for 60 s at 200 W. Cell debris was removed by centrifugation (24,000 × *g*, 20 min, 4°C) and EDTA was removed by dialysis against PBS (pH 7.4) at 4°C overnight. H22(scFv)-MAP was purified by immobilized metal-ion affinity chromatography (IMAC) on an Äkta Purifier System (GE Healthcare, Little Chalfont, UK) using IMAC Sepharose 6 Fast Flow as resin and an XK16-20 column (GE Healthcare). Preparative size exclusion chromatography (SEC) was carried out on an Äkta Purifier System (GE Healthcare) using an XK16-70 column packed with Superdex^TM^ 75 (GE Healthcare), with PBS as the mobile phase (pH 7.4) at a flow rate of 1.0 ml/min. The purity of H22(scFv)-MAP was determined by SDS-PAGE followed by staining with Coomassie Brilliant Blue and its identify was confirmed by western blotting. The protein concentration was determined by densitometric analysis of stained gels using AIDA software and was verified using the Bradford assay (Bio-Rad, Munich, Germany). Bovine serum albumin standards were used for calibration.

### Analysis of cell phenotypes

The phenotypes of the isolated cells were determined using labeled antibodies and flow cytometry. Double staining for CD33 and CD64 was carried out on leukemic material, and monostaining for CD14, CD64, CD163, CD200R, CD206, CD273, and CD301 was carried out on healthy material. Briefly, we incubated 2 × 10^5^ cells with 125 ng of anti-human CD33[P67.6]-APC, anti-human CD206[15–2]-PE, anti-human CD301[H037G3]-APC, anti-human CD163[GHI/61]-PE, anti-human CD273[MIH18]-PE, anti-human CD200R[OX-108]-PE, anti-human CD14[63D3]-PE, or anti-human CD64[10.1]-PE (all from eBioscience, Frankfurt, Germany) in 1% (v/v) human antibody-binding serum (BioChrom) for 30 min on ice in the dark, followed by washing with PBS. The corresponding mouse IgG1[MOPC-21] antibody (Biolegend, San Diego, CA, USA) with either a phycoerythrin (PE) or allophycocyanin (APC) label was used as the corresponding isotype control. Finally, the cells were washed twice with PBS and analyzed on a FACSCalibur flow cytometer (Becton Dickinson, Heidelberg, Germany) with Flowing Software v2.5.1.

### Binding analysis by flow cytometry

The cell-binding activity of Mock-MAP (non-binding control), H22(scFv)-ETA’ and H22(scFv)-MAP was analyzed by flow cytometry. We incubated 2 × 10^5^ cells with 200 ng of each construct in PBS (pH 7.4) containing 1% (v/v) human antibody-blocking serum for 30 min on ice followed by washing with PBS. Because each construct contained a His_6_-tag, fluorescence staining was carried out using 100 ng of an anti-His_5_ Alexa Fluor 488 antibody (Qiagen, Hilden, Germany) for 30 min on ice in the dark. Finally, the cells were washed twice with PBS and analyzed on a FACSCalibur flow cytometer.

### Apoptosis assay

An Annexin V/PI assay was used to determine the pro-apoptotic effect of H22(scFv)-MAP. We incubated 5 × 10^5^ cells per ml with 200 nM H22(scFv)-MAP or 200 nM H22(scFv)-ETA’ or 200 nM paclitaxel (Sigma Aldrich, Steinheim, Germany) in a 24-well plate (Greiner Bio-One GmbH, Frickenhausen, Germany) for 12 h at 37°C, 5% CO_2_ and 100% humidity. We used the anti-CD30 construct Ki4-MAP (200 nM) as the non-binding Mock-MAP control [20]. After incubation, the cells were washed twice with Annexin V buffer (pH 7.4) and stained with Annexin V–eGPF (produced by Anh-Tuan Pham, Fraunhofer IME, Aachen) in Annexin V binding buffer (10 mM HEPES/NaOH, pH 7.4), 140 mM NaCl, 2.5 mM CaCl_2_) for 30 min at room temperature. Finally, the cells were washed carefully as described above, resuspended in Annexin V buffer containing 10 μg/ml PI and analyzed by flow cytometry using a FACSCalibur flow cytometer.

### *In vitro* polarization of macrophages

Freshly-isolated PBMCs were cultured at a concentration of 5 × 10^5^ cells per ml for 48 h in complete medium supplemented with the appropriate polarization stimulus. Medium only was used for non-polarized M0 cells, 100 U/ml human IFNγ (Peprotech, Hamburg, Germany) was used for M1 cells, and 20 ng/ml IL-4 (Peprotech) was used for M2 cells. Sterile serum from patient AML M4 (I) was used to induce the TAM phenotype. The added serum amount (60 μl/ml) resulted in a final concentration of 20 ng/ml IL-4. No serum-mediated toxicity was observed in the TAM culture. Thereafter, macrophages were used for phenotyping or targeting experiments.

## SUPPLEMENTARY MATERIAL FIGURES


