# Supplementary material for: CD64-directed microtubule associated protein tau kills leukemic blasts ex vivo
Source: Oncotarget. 2016 Aug 24;7(41):67166–74. doi: 10.18632/oncotarget.11568 (PMC5341865; doi:10.18632/oncotarget.11568)
Supplement: Supplementary file 1 [file oncotarget-07-67166-s001.pdf]

CD64-directed microtubule associated protein tau kills leukemic blasts *ex vivo*

SUPPLEMENTARY FIGURES LEGENDS

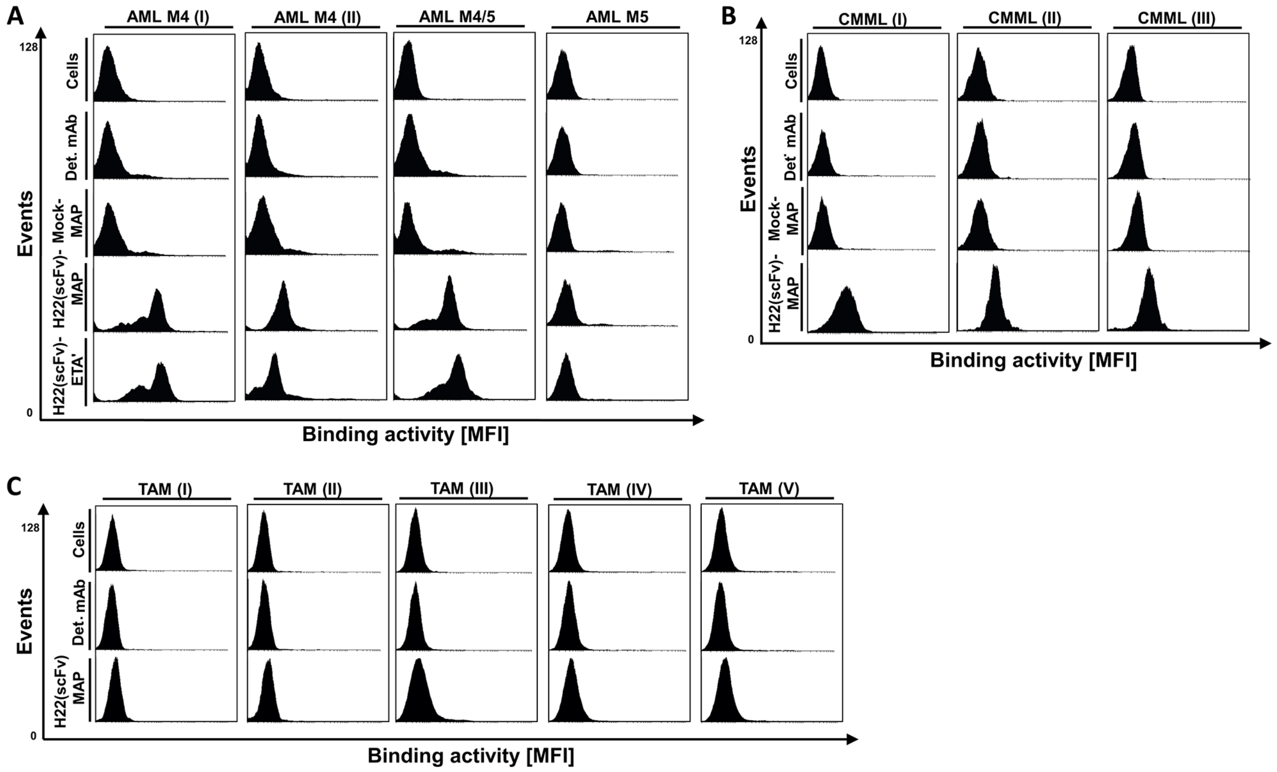

Supplementary Figure S1: H22(scFv)-MAP binds efficiently CD64+ leukemic blasts *ex vivo*. Binding of H22(scFv)-ETA' and H22(scFv)-MAP to primary CD64+ leukemic blasts from four AML patients **A.** three CMML patients **B.** and healthy PBMCs polarized to TAMs (n=5) **C.** Binding was detected by flow cytometry (gated on the ~90% viable population) using the non-binding construct Mock-MAP (anti-CD30) or the detection mAb alone as controls. Binding activity was detected using a monoclonal anti-His5 antibody labeled with Alexa 488. Successful binding is indicated by a population shift towards higher mean fluorescence intensity (MFI). Abbreviations: Det. mAb, detection monoclonal antibody alone.

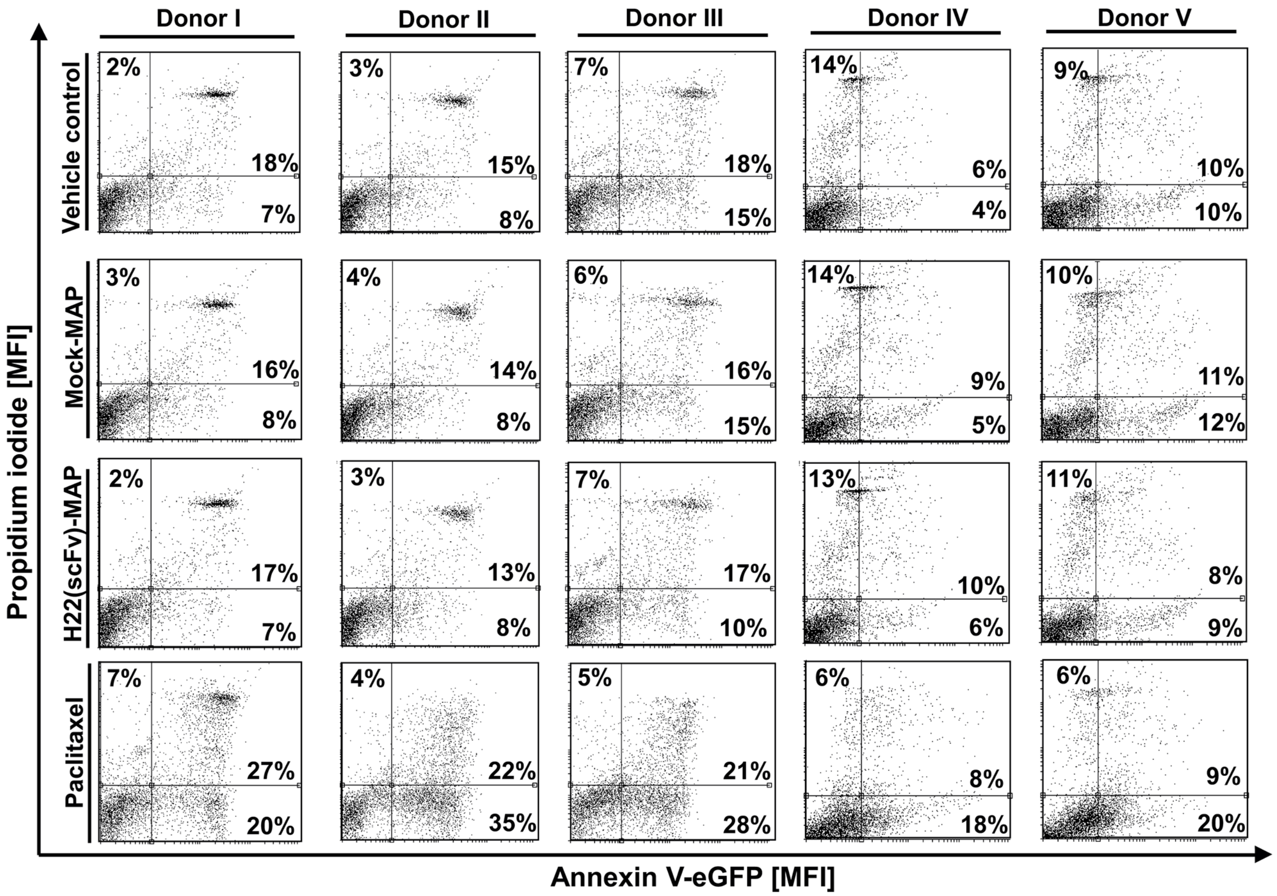

Supplementary Figure S2: H22(scFv)-MAP spares PBMCs derived from healthy blood. PBMCs derived from healthy blood donors were treated ex vivo with 200 nM H22(scFv)-MAP, Mock-MAP or paclitaxel for 12 h. Thereafter, the cells were stained with Annexin V-eGFP and PI. Dot blots (ungated) show the specific pro-apoptotic effect of paclitaxel compared to Mock-MAP and H22(scFv)-MAP (both lacking any cytotoxicity). The experiment was carried out in duplicate.

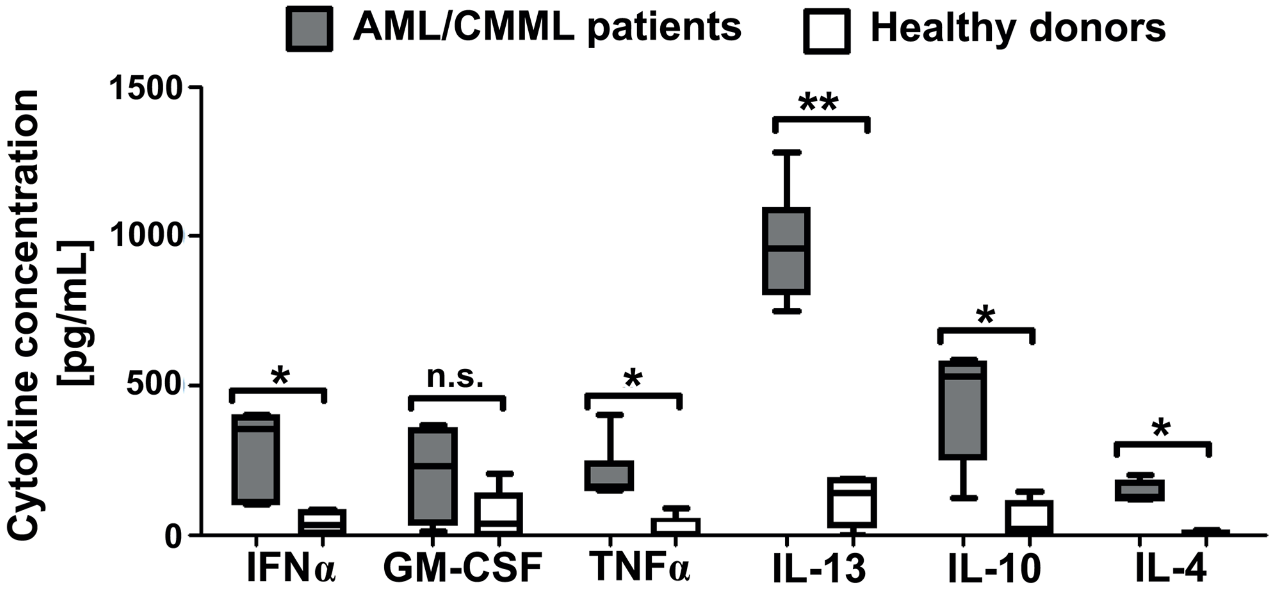

Supplementary Figure S3: Predominance of anti-inflammatory cytokines in serum from leukemia patients compared to healthy blood donors. The levels of pro-inflammatory cytokines (IFNα, GM-CSF and TNFα) and anti-inflammatory cytokines (IL-13, IL10 and IL-4) were measured in serum from six of the seven leukemia patients (2x AML M4, 1x AML M4/M5 and 3x CMML) and five healthy individuals using the FlowCytomix™ Multiple Analyte Detection System (eBioscience, Frankfurt) according to the manufacturer's instructions. Data are presented as box and whiskers plots with the interquartile range and minimum/maximum values indicated by the error bars. Statistical analysis was carried out using the non-parametric one-way ANOVA test (Kruskal-Wallis test by ranks): $^{*}p \leq 0.05$, $^{**}p \leq 0.01$.

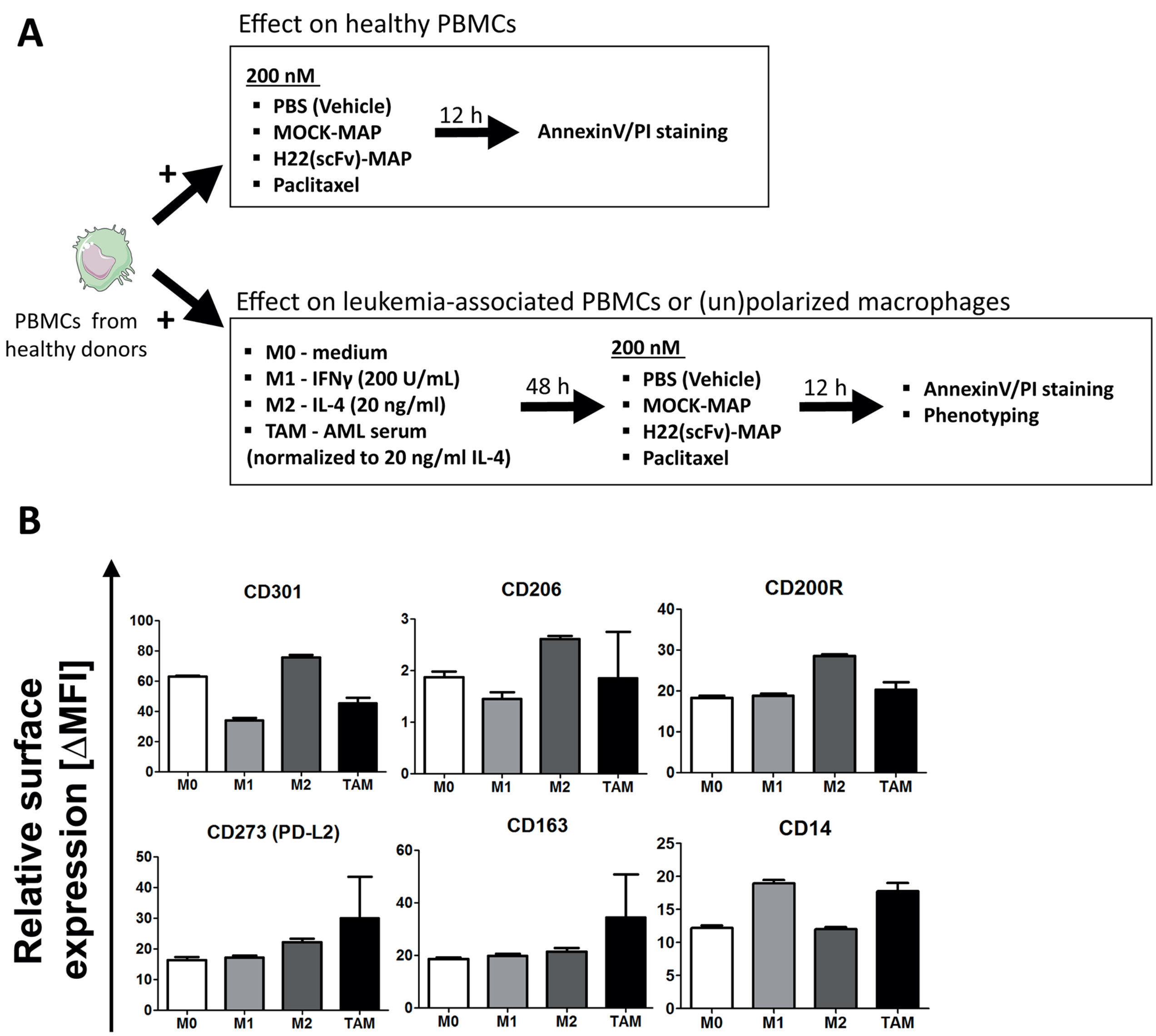

Supplementary Figure S4: Experimental setup using PBMC-derived macrophages from healthy donors. PBMCs were isolated from healthy blood donors and either directly used for targeting experiments or polarized to M1, M2 or TAM before targeting **A.** The M0 cells were medium conditioned macrophages, the M1 cells were induced using 100 U/ml human IFNγ, and the M2 cells were induced using 20 ng/ml IL-4. Sterile serum from patient AML M4 (I) was used to induce the TAM phenotype. The added serum was adjusted to a final concentration of 20 ng/ml IL-4. **B.** The expression of cell-surface receptors on macrophages was analyzed by flow cytometry. The results are normalized to the isotype control and are presented as a relative surface expression level.
